# The impact of social-environmental factors on IQ in syndromic intellectual developmental disabilities

**DOI:** 10.1017/cts.2024.510

**Published:** 2024-04-05

**Authors:** Walker S. McKinney, Desireé N. Williford, Leonard Abbeduto, Lauren M. Schmitt

**Affiliations:** 1 Department of Behavioral Medicine and Clinical Psychology, Cincinnati Children’s Hospital Medical Center, Cincinnati, OH, USA; 2 Department of Pediatrics, University of Cincinnati College of Medicine, Cincinnati, OH, USA; 3 MIND Institute, University of California Davis, Sacramento, CA, USA; 4 Department of Psychiatry and Behavioral Sciences, University of California Davis, Sacramento, CA, USA

**Keywords:** 22q11.2 deletion syndrome, cognitive development, developmental disabilities, Down syndrome, economic stability, environment, fragile x syndrome, genetic syndrome, home environment, intellectual developmental disabilities, IQ, parental stress, social determinants of health, social-demographic variables, syndromic IDD, socioeconomic status

## Abstract

Despite having the same underlying genetic etiology, individuals with the same syndromic form of intellectual developmental disability (IDD) show a large degree of interindividual differences in cognition and IQ. Research indicates that up to 80% of the variation in IQ scores among individuals with syndromic IDDs is attributable to nongenetic effects, including social-environmental factors. In this narrative review, we summarize evidence of the influence that factors related to economic stability (focused on due to its prevalence in existing literature) have on IQ in individuals with syndromic IDDs. We also highlight the pathways through which economic stability is hypothesized to impact cognitive development and drive individual differences in IQ among individuals with syndromic IDDs. We also identify broader social-environmental factors (e.g., social determinants of health) that warrant consideration in future research, but that have not yet been explored in syndromic IDDs. We conclude by making recommendations to address the urgent need for further research into other salient factors associated with heterogeneity in IQ. These recommendations ultimately may shape individual- and community-level interventions and may inform systems-level public policy efforts to promote the cognitive development of and improve the lived experiences of individuals with syndromic IDDs.

## Introduction

Intellectual developmental disabilities (IDDs), characterized by substantial limitations in intellectual and adaptive functioning, occur in about 1% of the general population [1]. Approximately 40–60% of IDDs have an identifiable genetic cause [2,3], with a subset of these IDDs considered syndromic based on the co-occurrence of other clinical features (e.g., facial dysmorphology) [4]. Marked differences in intellectual functioning (hereafter referencing IQ scores, the most common and well-validated index of intellectual functioning) in individuals with syndromic IDDs are driven by a highly penetrant genetic variant; however, the variant does not always necessitate a diagnosis of IDD as IQ scores in the average range are sometimes observed within a given syndrome [5]. Although the average range of full-scale IQ (FSIQ) scores differs between syndromes (Table 1), each syndrome’s respective genetic variant generally has a pronounced impact on IQ and serves as the primary contributor to the IDD phenotype.

**Table 1.** Means and standard deviations of full-scale IQ (FSIQ) for common syndromic forms of intellectual developmental disorder

Syndrome	FSIQ^†^ Mean (SD)	Reference
16p11.2 deletion syndrome	86 (15)	Moreno-De-Luca *et al*., 2015 [6]
Cornelia de Lange syndrome	42 (23)	Basile *et al*., 2007 [7]
De novo 22q11.2 deletion syndrome	75 (12)	De Smedt *et al*., 2007 [8]
Non-mosaic Down syndrome	52 (15)	Fishler *et al*., 1976 [9]
Fragile X syndrome (males only)	46 (9)	Dyer-Friedman *et al*., 2002 [10]
Prader–Willi syndrome	64 (12)	Whittington *et al*., 2004 [11]
Rubinstein–Taybi syndrome	55 (20)	Ajmone *et al*., 2018 [12]
Smith–Magenis syndrome	50 (13)	Madduri *et al*., 2006 [13]
Williams syndrome	55 (11)	Bellugi *et al*., 2000[14]

^†^ Many studies are unable to accurately assess FSIQ in patients with severe cognitive impairment due to floor effects and/or behavioral challenges during testing (often instead reporting developmental quotients or deviation IQ that relay only on raw scores). The means reported here, therefore, may reflect an overestimate of the syndrome “population level” FSIQ.

**Figure 1.**
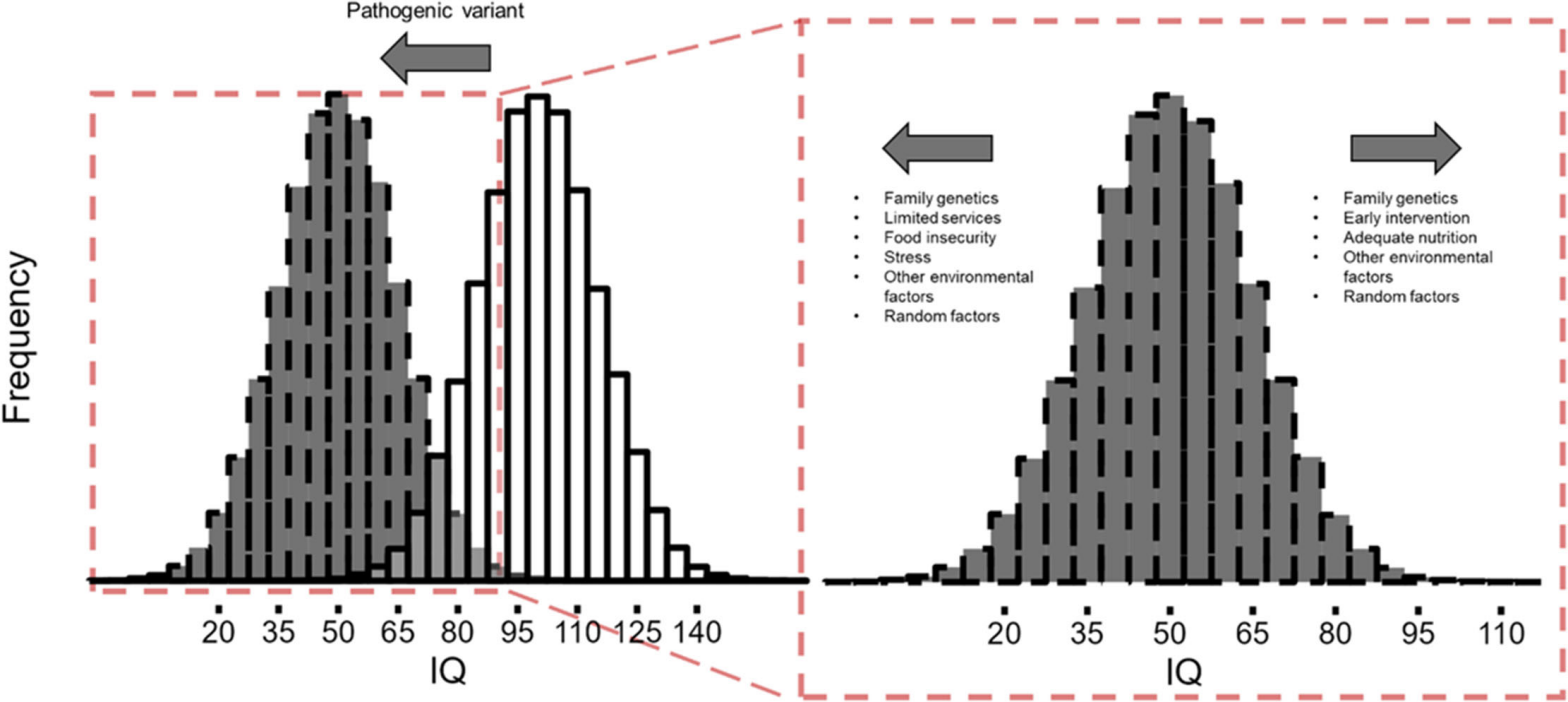
“Double hit” model. The pathogenic genetic variant contributes to the primary reduction in IQ in people with syndromic IDDs. Secondary genetic, environmental, epigenetic, and random factors contribute to smaller variations in IQ and result in a downshifted, but widely distributed, range of IQ scores. IQ = intelligence quotient; IDD = intellectual developmental disabilities.

Although the distribution of standardized intellectual abilities in people with syndromic IDDs is, by definition, down-shifted relative to the general population, mounting evidence suggests that IQs within the same form of syndromic IDD (e.g., fragile X syndrome, William syndrome, and Down syndrome) vary considerably across individuals. For some syndromes, the range of observed scores approximates a normal distribution after accounting for floor effects of standardized IQ measures [6,8,11,15–17]. Put another way, the population of individuals with the same form of syndromic IDD may have their own “syndrome-specific” down-shifted, nearly normal IQ distribution. This finding suggests that interindividual variability in IQ may be driven by a similar combination of genetic (both pathogenic and familial), environmental (shared and non-shared), interactive (e.g., epigenetic), and random factors, just as in the typically developing (TD) population [18]. We thus propose a “double hit” model that also incorporates measurement of nongenetic, environmental factors that may explain a significant amount of the variability in IQ among people with the same form of syndromic IDD (Fig. 1). This model specifies that the pathogenic genetic variant contributes to the initial large “hit,” or reduction, in IQ, whereas other familial genetic and nongenetic factors contribute to smaller but meaningful IQ differences in either direction.

Due to the high penetrance of the many genetic variants associated with IDDs, research into factors contributing to individual differences in IQ in syndromic IDDs has primarily emphasized genetic, molecular, or related biological factors. In contrast, relatively little attention has been paid to social-environmental factors (e.g., access to services, home environment, and nutrition) that might contribute to individual differences in IQ among individuals with syndromic IDDs. Two examples are considered to highlight the explanatory power of social-environmental differences. Only 30% of the variance in IQ among individuals with fragile X syndrome (FXS) is accounted for by familial genetic and molecular factors, including absence or reduction in fragile X messenger ribonucleoprotein (FMRP) production [10,15,19]. Likewise, 17.6% of the variance in IQ in individuals with 16-p11.2 deletion syndrome is accounted for by biparental IQ, or the average of maternal and paternal IQ scores [6]. Together, these findings indicate that nongenetic factors account for 70–80% of the remaining variance in IQ among individuals with these syndromic IDDs. Although these values underestimate the proportion of variance accounted for by genetic factors due to the underlying assumption that parent–child IQ correlations fully capture heritable genetic effects, these findings suggest that individual differences in IQ among individuals with syndromic IDDs, as in the TD population, are strongly influenced by nongenetic factors. Consistent with studies of physical health and other psychiatric and neurological conditions (e.g., Alzheimer’s)[20], expanding upon the behavioral genetics framework by incorporating the measurement of key social-environmental variables is clearly needed to comprehensively understand and support factors that promote cognitive development. This “bioecological model” has been successfully used to explain how social-environmental factors impact the heritability of intelligence [21,22], and such an approach may help clarify associations between pathogenic genetic variants, social-environmental factors, and IQ in syndromic IDDs.

Recent research has outlined methods for and stressed the importance of conducting more equitable IDD research that recognizes individual social-environmental differences that shape the daily experiences of individuals with IDDs[23]. The importance of this work has been further underscored by the research priorities of major funding organizations; for example, the National Institutes of Health (NIH) recently announced a new funding mechanism for research on the “exposome,” or “the totality of an individual’s exposures and the body’s response to them”[24]. This includes both physical and environmental (e.g., toxicants and pollutants) and social-environmental exposures (e.g., socioeconomic conditions, social capital, and discrimination). In the present narrative review, we meet these calls to action by summarizing evidence of the mechanisms through which select social-environmental factors may influence IQ in people with syndromic IDDs.

**Figure 2.**
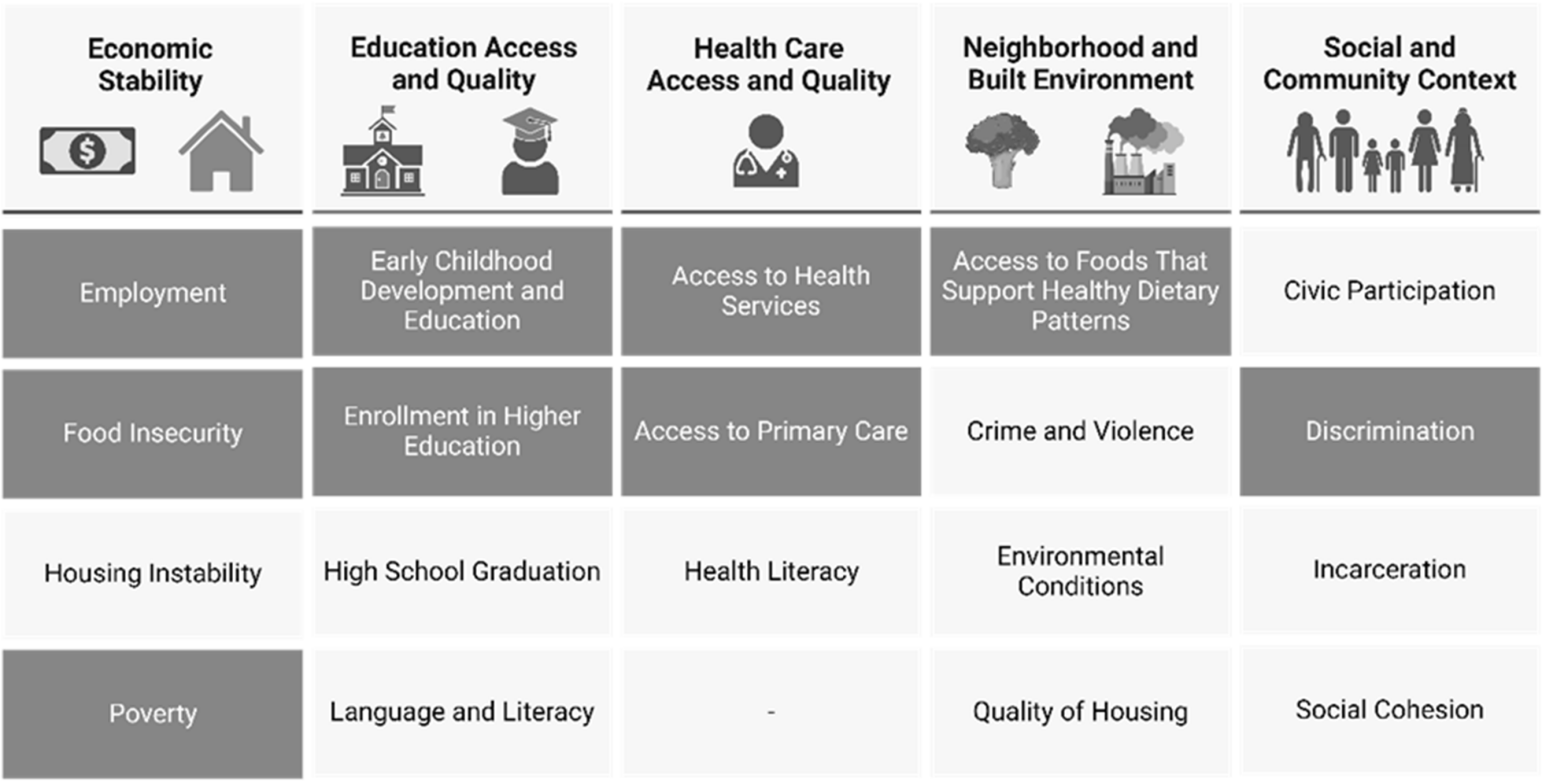
Social determinants of health identified by the US Department of Health and Human Services’ Healthy People 2030 Initiative [25]. Domains discussed in the present narrative review are depicted in darkened boxes. Figure created with BioRender.com.

Procedures for this narrative review included an unstructured review of the scientific literature for studies describing relations between social-environmental factors and IQ for people with syndromic IDDs as well as in other clinical and nonclinical populations when appropriate (WSM and LMS). Highly cited frameworks of social determinants of health (SDOH; “the conditions in the environments where people are born, live, learn, work, play, worship, and age that affect a wide range of health, functioning, and quality-of-life outcomes and risks;” US Department of Health and Human Services [25]) were also reviewed to identify multiple social-environmental domains that are likely relevant to IQ (Fig. 2). However, it should be noted that the influence of many SDOH on IQ has not been thoroughly examined in syndromic IDDs and the dearth of literature prohibited our ability to conduct a systematic review or meta-analysis. Based on available literature, we focus our review and resulting theoretical model on economic stability, or the ability to access resources (i.e., income/financial resources, food, housing, and employment) essential to one’s life and well-being.

Economic stability has been the predominant factor examined thus far in syndromic IDD research. As a set of SDOH that intersect with other important SDOH domains (e.g., healthcare and quality, education access and quality), clarifying the pathways through which it contributes to differences in IQ may help identify numerous cross-cutting targets for intervention in syndromic IDDs. The goals of the present manuscript are to summarize extant literature (i.e., narrative review) and articulate a theoretical conceptualization (i.e., formulating a conceptual model), two necessary initial steps for designing and conducting research aimed to understand and intervene upon these pathways. We focus on IQ as an outcome in our model because it has been the primary outcome in the majority of studies examining relations between clinical outcomes and social-environmental factors in individuals with syndromic IDDs. Still, we acknowledge the relatively greater importance placed on IQ in the medical literature and in diagnostic processes more generally, compared to other undervalued personal characteristics (e.g., self-determination and adaptive behaviors). We also acknowledge the known cultural biases in IQ test norming[26]. However, we believe the most practical first step is to understand the contributors to differences in IQ given the state of the literature as well as the robust positive associations between IQ and independent living, other cognitive abilities (e.g., executive functions), competitive employment, and quality of life in individuals with IDDs[27–31]. We also aim to provide guidance for researchers, reviewers, and journal editors by identifying key research priorities: namely, other SDOH that research in the TD population suggests are relevant to cognitive development, but which have been largely unexplored in individuals with syndromic IDDs. Through our identification of salient SDOH that most strongly influence cognitive development in syndromic IDDs and our articulation of an overarching conceptual model linking SDOH to cognitive outcomes, we aim to guide research and practice that may identify innovative, multilevel (e.g., individual and community) intervention targets and inform systems-level advocacy and sociopolitical change.

## Pathways through which economic stability influences cognitive development

Although economic stability has been shown to be an important domain within SDOH frameworks, it should be noted that the factors which comprise economic stability vary across models and the scientific literature. As defined by the US Department of Health and Human Services (US HHS) Healthy People 2030 Initiative, economic stability reflects a person’s access to economic resources, including employment opportunities, quality food and nutrition, safe and affordable housing, and a livable income [25]. Conversely, Hill and colleagues identify three domains of economic stability – income and employment stability, family stability, and benefits stability – which collectively support stability in housing, childcare, and access to healthcare [32]. Other researchers use the related term “economic security” to reflect income stability, medical spending, and accumulated wealth that protects families from large, unexpected expenses or loss of income [33]. The dearth of research on the influence of many of these economic components on the IQ of individuals with syndromic IDDs precludes the use of a singular organizational framework or definition in this review. Therefore, while we acknowledge the importance of each of the economic factors, we focus our discussion on those areas that have been explicitly studied in syndromic IDDs or the general population and demonstrate associations with IQ.

### Access to care

**Figure 3.**
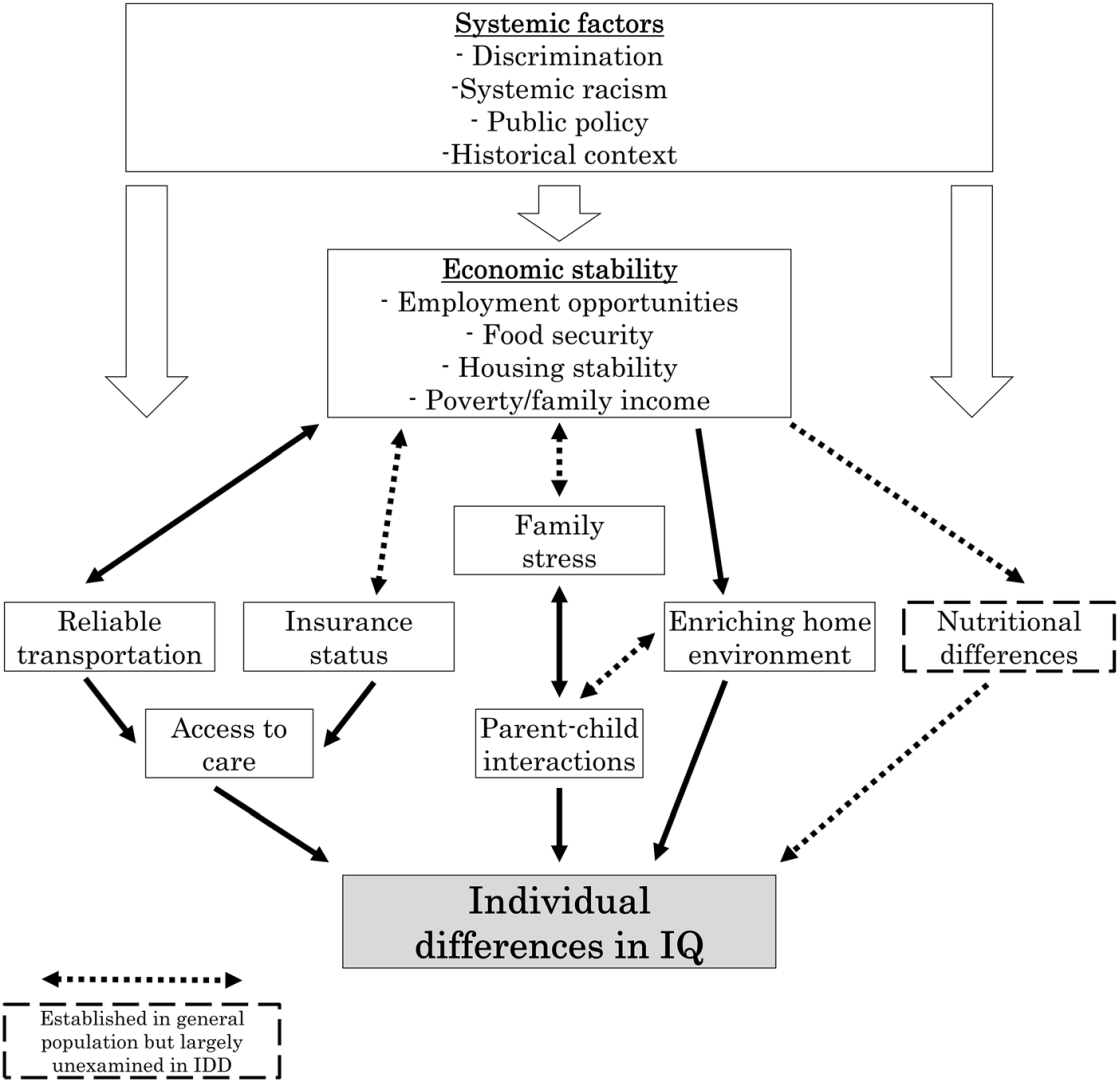
Potential pathway model for the impact of economic instability on IQ in individuals with IDDs. Note that many effects are bidirectional (e.g., economic instability limits access to reliable transportation which further fuels economic instability). Solid boxes and arrows reflect factors and pathways, respectively, that have been examined in individuals with syndromic IDDs. Dashed boxes and arrows reflect hypothesized factors and pathways that are presently unexplored in syndromic IDDs. IQ = intelligence quotient; IDD = intellectual developmental disabilities.

We propose that aspects of economic stability influence IQ in individuals with syndromic IDDs through multiple bidirectional, indirect, and interrelated pathways (Fig. 3). First, economic stability may account for some of the variability in IQ among individuals with syndromic IDDs due to the increased access to diagnostic, intervention, and educational services often afforded by higher income (Fig. 3, left side). Families experiencing poverty may face many barriers to obtaining services, including difficulties with payment and/or reliable transportation, which impede access to early diagnostic assessment and intervention [34,35]. Similar barriers frequently prevent uninsured or underinsured families from accessing well-child visits [36] that typically serve as the initial referral source for state early intervention programs funded by the Individuals with Disabilities Education Act (IDEA). Limited access to these services may in turn impede gains in IQ fostered by these programs [37–40] and could account for interindividual differences in IQ among people with syndromic IDDs. Limited service access also may stem from limited service availability, which can be driven by factors related to rurality and/or low provider/service density rather than factors exclusively related to economic stability. In fact, recent work demonstrates that service access is as limited for individuals with IDDs living in nonmetropolitan areas as it is in the general population [41]. Furthermore, access to syndromic IDD clinics, where providers with specialized training dedicated to a specific syndromic IDD deliver specialized care, is limited by the scarcity of these clinics and their location almost exclusively in major metropolitan areas. Thus, higher family income facilitates access (i.e., travel and affordability of services) to these specialty clinics [42,43] and may promote cognitive gains through specialized service access.

Higher income also may facilitate increased educational opportunities for parents (e.g., increased college attendance) [44] and children (e.g., greater availability and higher quality of childcare, school, and behavioral intervention programs) [45,46] that drive cognitive growth. For example, access to higher-quality preschool programming and early behavioral intervention improves IQ in children with and without IDDs [47,48]. Family income also is positively associated with subjective and objective indicators of behavioral health service quality [49,50], indicating that the quality of services, in addition to service access, may mediate the association between economic factors and IQ.

Importantly, the impact of economic stability on service access and subsequent changes in IQ also intersects with systemic racial and ethnic discrimination and bias: people from marginalized racial and ethnic backgrounds are disproportionately more likely than others in the population to experience poverty and to be un- or under-insured, ultimately limiting access to these high-quality services [36,51]. As one example in the context of syndromic IDDs, Black families face systemic barriers in accessing specialized neurodevelopmental healthcare services [52] and, therefore, they receive their child’s diagnosis of FXS later in life [53]. This IDD-specific finding is consistent with the broader pattern of lower rates of confirmed genetic diagnoses in people from marginalized racial and ethnic backgrounds linked to implicit bias in providers, limited availability of specialized genetic diagnostic services, and higher rates of inconclusive genetic testing results [54]. These barriers delay access to treatment options that are more readily available following receipt of a medical diagnosis (e.g., targeted medical management for issues known to be associated with the specific genetic variant) that would foster positive cognitive and adaptive development.

### Home environment, home enrichment, and family stress

Economic instability and the stress it creates may significantly influence the family home environment, including home enrichment and caregiver–child interactions, both of which impact cognitive development (Fig. 3, center). The quality of the family’s home environment promotes cognitive development through the provision of enriching learning opportunities (e.g., trips outside the home, access to early educational materials, and role-playing toys) and thus may account for interindividual differences in IQ in individuals with syndromic IDDs. Several studies of FXS have examined the impact of the home environment on cognitive outcomes in children. For example, improved quality of the home environment is associated with higher IQ, although this association is substantially stronger in boys with FXS than it is in girls with FXS [10]. Similarly, a more enriching home environment is associated with greater adaptive skills in boys, but not girls, with FXS [55]. Sex (as assigned at birth) differences in the association between home environment and developmental outcomes may be related to sex-associated genotypic and phenotypic differences specific to FXS, wherein females with FXS typically have less pronounced deficits due to X-inactivation [56]. Furthermore, Glaser and colleagues hypothesized that families of boys with FXS, relative to families of girls with FXS, may be more likely to enrich their home environment out of necessity due to their boys’ increased support needs [55]. In other words, differences in the home environment may have less of an impact on females with FXS because their stronger cognitive and adaptive skills, relative to males with FXS, may not necessitate as significant of environmental intervention (e.g., behavioral therapy and changes to home structure). Future work examining the association between home environment and IQ, therefore, should be mindful of the moderating impact of biological factors, including sex assigned at birth (especially if related to phenotypic differences for the syndrome of interest), and cognitive and adaptive behavioral strengths.

Economic instability also contributes to family stress, which may influence IQ through several pathways. The direct impact of family stress due to economic hardship likely begins prenatally, as maternal stress is associated with birth complications (e.g., preterm birth and low birth weight) linked to developmental delays and lower IQ [57,58]. One neurodevelopmental mechanism underpinning lower IQ may be reduced postnatal brain volume resulting from maternal stress and socioeconomic disadvantage occurring during pregnancy [59]. Long-term physiological stress or “allostatic load,” also may exacerbate the detrimental effects other factors have on IQ, as has been demonstrated in studies of environmental pollutants [60]. The influence of economic stability on family stress and child IQ likely continues postnatally. Heightened parental stress and the increased parental attention required to fulfill the family’s basic needs may influence parent–child interactions, which play a crucial role in cognitive development [61,62], as demonstrated in the general population [63]. For example, caregivers of youth with IDDs are more likely to experience unemployment [64], possibly due to barriers related to the increased caregiving needs inherent in raising a child with IDD (e.g., safety monitoring, attending frequent appointments, limited availability of other skilled caregivers, or respite care). Unemployment subsequently contributes to greater family stress and may influence the nature of caregiver–child interactions. Consistent with this hypothesized pathway linking parent–child relational factors, stress, and child IQ outcomes, several studies indicate that lower family distress and closer parental relationships are associated with greater verbal and nonverbal IQ in individuals with FXS [65] as well as stronger verbal skills in individuals with FXS or DS [66]. Specifically, del Hoyo Soriano and colleagues found that both closer mother–child relationships and lower maternal distress predicted greater IQ scores in a cross-sectional sample of youth with FXS, whereas genetic/biological factors (e.g., higher FMRP levels and lower proportion of affected chromosomes) predicted longitudinal gains in nonverbal IQ [65]. Similarly, a more responsive parenting style is associated with stronger language skills in individuals with FXS [67].

The impact of caregiver stress due to economic instability on child IQ may be especially pronounced for verbal skills due to the unique impact caregiver stress has on caregiver–child interactions important for language development [68,69]. Specifically, more frequent reciprocal parent–child verbal interactions promote language development and verbal problem-solving through parental scaffolding and modeling [68–72]. These interactions are amenable to intervention in caregivers of youth with neurodevelopmental disabilities, including FXS and other forms of syndromic IDDs [73–75], highlighting the potential efficacy of caregiver-mediated interventions for improving IQ given the malleability of caregiver behaviors through behavioral interventions.

Importantly, family stress is affected by other broader sociocultural and systemic factors, such as racial and ethnic discrimination. Discrimination-induced stress begins to influence neurodevelopmental and health outcomes, such as birth weight, that are linked to differences in IQ prenatally [76,77] and therefore may have long-lasting impacts on cognition in individuals with syndromic IDDs whose birth mothers hold marginalized racial and ethnic identities. Further, some stress-related pathways may be cyclical. For example, marital stress in families of youth with neurodevelopmental disabilities is strongly influenced by economic disadvantage and is linked to changes in family makeup (e.g., divorce and shifts to a single-parent household) [78], which may further contribute to family-level stress due to increased caregiving demands [63]. These added demands may subsequently influence caregiver–child interactions.

It is critical that measures of the quality of the home environment capture differences in both home enrichment and parent–child interactions. The Home Observation for Measurement of the Environment (HOME), for example, includes a qualitative assessment of the availability of enriching toys and media, frequency and types of visits outside the home, home factors (e.g., light and cleanliness), parental interactions with the child (e.g., discipline, verbal communication, and keeping child in view), child responsibilities and expectations, and other indicators of a safe and nurturing home [79]. It also is important to recognize that other social-environmental factors may impact home environments and indirectly influence cognitive development. For example, caregiver education, extended family support, mental health literacy, and incarceration collectively or in isolation influence the home environment and parent–child interactions [80–83]. The influence of these factors is largely unexplored in syndromic IDDs, and research into these areas is needed to clarify additional targets for multilevel and systemic interventions, including public policy solutions. Ultimately, the collective influence of these home factors occurring across multiple levels highlights the importance of individual-, family-, and systems-level strategies that foster economic success, reduce family stress, and promote positive caregiver–child interactions that contribute to gains in IQ among individuals with syndromic IDDs.

### Nutritional differences

Economic stability may influence differences in IQ in individuals with syndromic IDDs due to the impact of high-quality nutrition and food security on neurodevelopment (Fig. 3, right side). As with stress, the influence of nutrition begins prenatally: better maternal nutrition during pregnancy is associated with greater childhood IQ at 8 years of age in the TD population [84]. Adequate maternal nutrition (e.g., micronutrient consumption and protein intake) improves birth outcomes such as birth weight [85] and, through positive effects on neurodevelopment, may contribute to greater IQ. Access to healthcare and health literacy both likely intersect with prenatal nutritional outcomes, such that mothers experiencing barriers to accessing prenatal healthcare appointments (e.g., transportation [86] and insurance [87]) are less likely to receive nutritional guidance during their pregnancy. Although no study to date has specifically looked at postnatal relations between food security and IQ in syndromic IDDs, food insecurity is known to increase the likelihood of developmental delay even after accounting for household income [88]. Infantile malnutrition also is associated with lower IQ scores in childhood and adulthood [89,90]. We hypothesize that these same relations exist for individuals with syndromic IDDs, but this is an important area of consideration for future research.

### Bidirectional effects

Many of the above-hypothesized pathways influencing IQ likely are bidirectional, and many factors are interrelated and intersectional (Fig. 3) [91,92]. For example, greater food insecurity is associated with higher healthcare expenditures, likely due to the negative impact of food insecurity on health, and higher healthcare expenditures are associated with a greater likelihood of being food-insecure, likely due to income previously allotted for food instead being allotted to pay for medical care [92]. Similarly, caregiver stress driven by economic instability may cyclically contribute to greater economic instability due to the impact of caregiver mental health on employment and income, although it is important to note that socioeconomic disadvantage itself uniquely limits neurodevelopmental outcomes (i.e., above and beyond its role in generating stress) [59]. Longitudinal studies indicate that suboptimal mental health symptoms predict and are predicted by unemployment and loss of income [93–95]; in other words, it is harder to find and maintain employment while under significant stress and while living with anxiety and depression [95], and difficulties finding and maintaining employment subsequently contribute to stress, anxiety, and depression [96,97]. Together, these findings suggest that a constellation of interrelated pathways, including the bidirectional impact of unemployment/underemployment and loss of income on access to intervention services, stress and mental health, and home environment, collectively contribute to individual differences in IQ among individuals with syndromic IDDs. The impact of individual and collective facets of economic stability on IQ is wide-ranging and likely transdiagnostic, highlighting the critical importance of developing interventions and public policy solutions that drive systems-level change across and within social-environmental contexts and which operate independent of an individual’s specific genetic diagnosis.

## Evidence for the association between economic stability and IQ in syndromic IDDs

Although possible mechanisms extend beyond those described in this article, the developmental benefits (e.g., higher IQ) of economic stability through the mechanisms described above are well established in the general population. However, these pathways have not yet been comprehensively examined in individuals with syndromic IDDs. However, several key studies have examined relations between IQ and global measures of economic stability that correlate with factors more directly linked to differences in IQ in individuals with syndromic IDDs, although results have been inconsistent [10,98–102]. Equivocal findings from studies investigating the association between child IQ and economic factors in syndromic IDDs are likely driven by (1) study-specific measurement differences, (2) the IQ domain assessed (e.g., verbal, nonverbal, or full-scale), and (3) age and developmental effects.

These studies have primarily used four different indices to measure economic stability, each with relative strengths and weaknesses. First, economic stability has been directly assessed using continuous measures of adjusted gross family income controlling for area cost of living [10]. This is a useful measure due to ease of interpretation and standardization across regions but fails to account for important contextual factors like accumulated wealth, employment stability, and job benefits. Second, economic stability has been assessed using measures of socioeconomic status (SES) and conversion of parental occupation to a standardized SES index score [98]. These index scores are useful because, relative to measures of gross annual household income, they allow for the more stable and reliable comparison of income across regions and countries while incorporating more abstract qualities such as occupational prestige and socioeconomic mobility [103]. However, their scoping nature impedes interpretation of the specific mechanisms through which economic stability influences IQ. Third, several studies have relied on parental education (typically as a proxy for parental IQ), which may clarify the association between economic stability and child IQ due to the robust, albeit indirect, association between household income and access to postsecondary education [44]. Although parental education is easily assessed and reliably associated with income, it is heavily influenced by and confounded with familial genetic factors [104]. Last, economic stability has been measured as part of a composite sociodemographic family variable comprised of factors such as parental IQ, household income, and parental education (e.g., Kover *et al*., 2013) [99]. As with standardized SES indices, these composite variables may capture multiple facets of socioeconomic advantage but lack specificity. Ultimately, a combination of measures is needed to comprehensively capture individual differences in economic stability while also identifying mechanistic pathways amenable to intervention.

Inconsistent findings also may be attributed to the association between economic stability and the diverse range of cognitive domains reflected in IQ scores. Malich and colleagues [100], quantifying SES as a composite of two six-point scales measuring paternal occupation and maternal education, demonstrated positive associations between parental SES and child FSIQ in youth with Prader–Willi syndrome. Other studies have similarly reported associations between greater parental education and greater child IQ in youth with 22q11.3 deletion syndrome [101] and adolescents with Down syndrome (DS) [105]. In contrast, multiple studies of FXS have failed to find a significant association between family-level economic factors and IQ, although this association has primarily been assessed using measures of nonverbal IQ [10,99,102]. Specifically, Dyer-Friedman and colleagues [10] found no significant association between household income and child IQ in youth with FXS. Similarly, Skinner and colleagues [102] similarly found no association between nonverbal IQ (as measured by the Leiter-R) and maternal education in FXS. Kover and colleagues [99] also found no significant association between maternal IQ, family income, and parental education (combined into a single composite variable) and child nonverbal IQ (Leiter-R) in adolescents with FXS. These latter findings suggest that the impact of economic stability on IQ may be more pronounced for measures of verbal IQ than other domains of cognition. Indeed, there is evidence that associations between SES and IQ are stronger for verbal relative to nonverbal skills in youth with 22q11.3 deletion syndrome [101] and DS [105]. This echoes findings from Del Hoyo and colleagues [66] that higher maternal education is associated with greater increases in verbal abilities (i.e., conversational talkativeness) in adolescents with FXS and DS. Relative to nonverbal IQ, verbal IQ and language skills are more strongly influenced by environmental factors [106,107], which may make them more susceptible to the influence of economic stability and related factors described above. Together, these findings indicate that economic stability accounts for some individual differences in IQ among people with syndromic IDDs, though this association may be exclusive to, or more pronounced for, verbal IQ.

Last, differences in results among these studies also may be due to sample characteristics, especially differences in age. In the general population, the heritability of IQ increases linearly with age from 41% in 9-year-old children to 66% in 17-year-old young adults [108]. Therefore, the impact of nongenetic factors, including social-environmental differences, may be amplified in early childhood. Relative to studies of the general population, few studies have examined how the heritability of IQ varies across development in individuals with syndromic IDDs. The pattern of increasing heritability of IQ with age observed in the general population appears to be present in some syndromic forms of IDDs, including DS [105]. However, individuals with other syndromic forms of IDDs, such as 22q11.2 deletion syndrome, show more consistent levels of heritability of IQ across childhood and adolescence [109]. Other studies examining the heritability of IQ among individuals with syndromic IDDs have examined relatively broad age ranges (e.g., 3–20 years) [100], finding moderate parent-proband IQ correlations, but have not yet explicitly assessed how this association varies across development. Studies examining the moderating effect of age on relations between social-environmental factors, parental IQ, and proband IQ in families of individuals with syndromic IDDs are needed to identify the periods most sensitive to targeted efforts intervening on social-environmental disadvantage.

## Social-environmental areas for future consideration

Thus far, we have focused on select, relatively well-researched areas for which there is the most support for their influences on IQ in individuals with syndromic IDDs. However, there are many SDOH that may contribute to differences in IQ but whose impact has not yet been examined in individuals with syndromic IDDs (see non-darkened boxes of Figure 2 and dashed pathways of Fig. 3). For example, social and community contextual areas, including social cohesion [110], discrimination [111,112], and incarceration [113,114], have been identified as salient factors contributing to cognitive outcomes in the TD population, but these have not been fully explored in individuals with syndromic IDDs.

Social cohesion at the individual level (e.g., the strength, density, and number of a person’s social relationships [115]) represents a promising area of study given the challenges many individuals with IDDs face in integrating into new neighborhoods and establishing and maintaining friendships, which results in high rates of loneliness [116,117]. Greater loneliness is associated with lower IQ in aging TD adults [110], possibly due to reduced cognitive stimulation available through social interaction, and this pattern, though largely unexplored, may hold in individuals with IDDs. At the level of the family unit, stronger social support is associated with lower parental stress and greater family adaptability in families of individuals with IDDs [118,119], as well as greater family hardiness and quality of life in families of autistic youth [120,121]. Associations between family support and positive quality of life outcomes may be due to greater availability of other family members and friends to assist with childcare in addition to the well-established social-emotional benefits (i.e., reduced stress) of having supportive relationships. Lower family stress may in turn support cognitive development through mechanisms described earlier (greater reciprocity of parent–child interactions and bidirectional positive impact on economic stability), though no studies to our knowledge have tested these mediating relationships.

Second, despite the high degree of stigma and discrimination experienced by individuals with IDDs and their family members [122], especially those who also hold marginalized racial and ethnic identifies [118,119,123–127], their impact on IQ and cognitive outcomes is not well understood in syndromic IDDs. The lack of research on these associations may be related to limited availability of validated self- or proxy-report measures of discrimination in individuals with IDDs, as self-report measures of discrimination are limited to individuals with mild to moderate IDD [128]. Despite limited availability of suitable self-report measures to assess discrimination, studies assessing other outcomes linked to cognitive development (e.g., health and quality of life) in people with IDDs from marginalized racial and ethnic backgrounds may help clarify the impact of discrimination. For example, non-White individuals with IDDs or other neurodevelopmental disabilities (e.g., autism) experience a lower quality of life on average than their White peers, in part due to a disproportionately limited number of decision-making opportunities [123,124]. Similarly, adults with IDDs who are from marginalized racial and ethnic backgrounds experience poorer health outcomes and/or face a greater number of barriers to accessing care compared to both White adults with IDDs as well as adults who are from marginalized racial and ethnic backgrounds without IDDs [127,129]. The multiplicative consequences of discrimination based on disability and racial/ethnic discrimination cut across these and other areas relevant to development, which may in turn drive divergent cognitive outcomes in individuals with syndromic IDDs. Increased stigma around IDD in some cultures also limits family belonginess and may subsequently impact the ability of an individual with syndromic IDDs to establish social relationships important for development, increase family stress, and limit access to educational and recreational opportunities (please see review for further discussion of this issue and possible interventions [130]). Ultimately, given the intersection of stigma and discrimination with most social-environmental issues [131], clinical studies incorporating the measurement of stigma and discrimination (both due to disability and other marginalized identities) should be prioritized.

The effects of racial and ethnic discrimination experienced by family members also may have an indirect impact on IQ in individuals with syndromic IDDs. As previously described, the negative impact of systemic racial and ethnic discrimination on IQ in individuals with syndromic IDDs may be partly attributable to prenatal exposure to stress [76,77], delayed access to services [52–54], poverty [51], and other factors. Discrimination individually directed toward family members also increases caregiver stress, which may in turn alter parent–child interactions that shape cognitive development and IQ, as noted above.

Last, the incarceration of family members of individuals with IDDs also may contribute to interindividual differences in IQ, but no studies to our knowledge have explicitly examined these relations in this population. Incarceration of a primary caregiver limits caregiving support and family income which subsequently increases family stress and financial hardship, especially in parents of children with greater healthcare needs such as those with syndromic IDDs [132,133]. Greater family stress and economic instability driven by incarceration may in turn hinder the cognitive development of individuals with IDDs through the pathways identified in Figure 2.

### Limitations

The present narrative review is an important first step to advance syndromic IDD literature, yet is not without its limitations. First, IQ is not the only area in which individuals with syndromic IDDs show large interindividual differences; however, we focused on IQ due to its emphasis in the existing literature. IDDs are characterized by limitations in adaptive behaviors (e.g., daily living skills), which also show large interindividual differences among people with syndromic IDDs [134]. Correlations between IQ and adaptive behaviors in individuals with IDDs are generally low [134,135], and it is not clear whether the same factors affecting IQ also affect adaptive skills. For example, a study of preschoolers exposed to the Flint water crisis suggests family SES disproportionately impacts IQ, whereas home environment disproportionately impacts adaptive behavior [136]. Consistent with this, in individuals with FXS, family income is not significantly associated with adaptive behavior after accounting for the quality of the home environment [55]. Together, these findings suggest that the quality of the home environment may mediate the impact SES has on adaptive development, although this pathway has not been explicitly modeled to our knowledge. Given the impact that adaptive behaviors have on independence and quality of life, research into factors that maximize adaptive development is needed. Moving beyond measures of IQ also is critical to avoid conflating meaningful differences in cognitive development resulting from malleable social-environmental factors with differences caused by known systemic cultural bias in test norming [26].

Second, we chose to focus our discussion on the impact of economic stability and related mechanistic pathways reflecting the greatest volume of research in these areas. We acknowledge that other key social-environmental factors, such as neighborhood crime or violence, housing quality, and social cohesion, are potentially important and have received limited attention in the literature, despite being strongly associated with key economic factors such as neighborhood income and poverty [137–139]. Studies assessing the association between additional SDOH factors and IQ are needed to fully understand ways to optimize outcomes for individuals with syndromic IDDs.

Finally, our narrative review is not exhaustive. We chose to pursue a narrative review over a systematic review or meta-analytic approaches due to the overall dearth of IDD-specific research in this area. We acknowledge that there may be studies of social-environmental factors as they relate to IQ not discussed in our narrative review. However, this choice allowed us to more readily integrate findings from the general population and broader neurodevelopmental disabilities (e.g., autism) literature into the proposed theoretical model on relations between social-environmental factors and IQ. The use of a narrative review allows us to build on these findings to discuss future directions for the field of IDD. We also acknowledge that by pursuing a narrative review, there is a greater risk for bias in the studies we have discussed. Our authorship team’s expertise is in FXS and DS (WSM, LA, and LMS), and we are more likely to draw on the literature in those areas. Ultimately, we believe our review reflects the preponderance of literature in this area because FXS and DS two of the most well-researched syndromic IDDs due to their prevalence and penetrance – FXS is the most common heritable cause of IDD and DS is the most common genetic cause of IDD. However, we have done our best to combat this bias by explicitly targeting studies of other forms of syndromic IDDs and by conceptualizing our model in collaboration with an author with expertise in public health and SDOH in pediatric populations outside of FXS and DS (DNW).

### Recommendations

As previously described [23], systemic changes are needed to advance IDD research, and we propose that research which promotes and clarifies relations between social-environmental factors and IQ in individuals with syndromic IDDs is one way to do so. Building upon the extant literature and our proposed theoretical model, we make three aligned recommendations for researchers, reviewers, and journal editors to propel the field and address current research gaps (Table 2).

**Table 2.** Recommendations to delineate relations between social-environmental factors and IQ in individuals with syndromic IDDs

Issue	Recommendation		
	Responsible party		
	*Journal editors*	*Reviewers*	*Researchers*
1. Infrequent reporting of basic demographic information	- Publish editorial board expectations regarding reporting of demographic in empirical articles	- Request demographic information when absent (e.g., race, ethnicity, primary language, SES, etc.)	- Measure and report basic demographic information
			- Prioritize inclusion of participants from groups historically excluded from and underrepresented in research
			- Establish field consensus on “gold-standard,” scalable measures of social-environmental factors
	- Promote studies of inclusive and representative samples		- Incorporate measures that target the mechanism through which social-environmental factors impact development
2. Limited understanding of relations between social-environmental factors and clinical outcomes in IDD	- Prioritize special issues and calls for articles examining these associations	- Encourage the addition of social-environmental information into models (even as supplemental material)	- Incorporate stepwise measurement of social-environmental factors into biobehavioral models
			- Consider forming an interdisciplinary team to leverage the expertise of related research fields
3. Insufficient research to use existing Social Determinants of Health frameworks	- Identify internal lists of reviewers with a strong understanding of this framework	- Become acquainted with existing frameworks	- Prioritize the study of domains whose impact on cognition is seldom studied in IDD (e.g., food insecurity, exposure to crime and violence, health literacy)
	- Provide guidelines for authors hoping to include this information in submissions		

SES = socioeconomic status; IQ = intelligence quotient; IDD = intellectual developmental disabilities.

#### Recommendation 1: Measure and report demographic information

Researchers should measure and provide more details about demographic factors, including information on race, ethnicity, recruitment catchment area, socioeconomic resources (e.g., income or, preferably, more comprehensive standardized measures such as the Child Opportunity Index or Area Deprivation Index), age of first diagnosis, insurance status, and receipt of services. Given the inconsistent reporting of this information in the neurodevelopmental and broader pediatric literatures [140,141], we encourage journal editors and reviewers to emphasize the importance of including this information and communicate previously published guidelines on terminology and language for authors to consider when reporting this data [142]. For example, multiple prominent medical, psychological, and IDD-adjacent journals require that authors report participants’ race, ethnicity, age, gender, and SES. It also is critical to extend these basic measures by also assessing factors and pathways thought to have a more direct impact on cognition, which may clarify the mechanisms through which social-environmental factors impact development in syndromic IDDs. For example, there is a rich literature on relations between family stress and child outcomes in caregivers of youth with IDDs [65,143,144].

As previously noted, studies assessing relations between economic stability and IQ in individuals with syndromic IDDs have measured economic factors using varying methodologies, with a general focus on household income or caregiver SES. As recommended by Kover and colleagues [99], the impact of broader economic stability on IQ requires adopting a comprehensive, standardized measure of economic opportunity, such as the International Socio-Economic Index [103], Child Opportunity Index [145], or the Area Deprivation Index [146], the latter two of which incorporate multiple measures of economic disadvantage (e.g., employment opportunities, housing quality, and income). The association between greater neighborhood-level deprivation and lower IQ is strong in the general population [147], but its relation with IQ has not yet been studied in individuals with syndromic IDDs. The Child Opportunity Index and Area Deprivation Index are worth incorporating in future studies of syndromic IDDs, especially given the low administrative and participant burden for their measurement (i.e., home address). Further, incorporating additional measures assessing the actual mechanisms (e.g., nutritional differences) through which these wide-reaching economic characteristics influence IQ will clarify the nature and malleability of these relations.

Researchers in this area also may consider incorporating well-validated measures of stress due to racism and discrimination, such as the Race-Based Traumatic Stress Symptom Scale [148], as stress due to racism and discrimination, rather than race or ethnicity itself, hinders developmental outcomes. In addition to reporting demographic information, research teams also should prioritize recruitment of groups historically excluded from and underrepresented in IDD research, including non-English-speaking families, families affected by poverty, and families holding marginalized racial and ethnic identities [149].

#### Recommendation 2: Assess relations between social-environmental factors and clinical outcomes

Researchers should focus on examining the relation between social-environmental factors and clinical outcomes (including IQ) in studies of syndromic IDDs to address current gaps in understanding, such as those identified in Figures 2 and 3. In practical terms, this entails incorporating social-demographic variables into models predicting clinical outcomes during their stepwise construction (e.g., Dyer-Friedman *et al*., 2002) [10] to determine the *additional* variance in clinical outcomes captured by social or environmental factors. Consider, for example, researchers studying the efficacy of a parent-mediated intervention on language development of youth with IDDs. Researchers typically examine how basic demographic characteristics, including child age or sex, explain differences in treatment response. However, there are a host of other social-environmental factors that likely influence treatment fidelity or response, including age of diagnosis (as a proxy for delayed access to care), parent education, family-level stress driven by economic instability, and incarceration limiting caregiver availability. An initial model assessing treatment response may include treatment condition (novel treatment vs. treatment as usual), child age, and child sex. Subsequent models may add these relevant social-environmental factors to assess their moderating impact on treatment response. In this example, researchers would then be able to assess whether families experiencing high stress due to economic factors are less likely to benefit from a parent-mediated treatment. Thus, incorporating these factors in models assessing treatment response could ultimately inform treatment decisions when the intervention is translated into community practice.

Conducting this work as part of interdisciplinary teams and using team science approaches will leverage the expertise of related fields of research (e.g., sociology, epidemiology, and economics) to better inform the development of individual, community, and systems-level interventions that target these pathways. We further encourage researchers to examine these relations even in studies of multiple forms of syndromic or non-syndromic IDDs, as these associations are likely transdiagnostic. However, no study to our knowledge has systematically examined the differential impact of social-environmental factors on IQ across multiple forms of IDDs. Although such studies are inherently difficult to conduct due to the rarity of syndromic IDDs, they may identify associations and intervention targets that vary across syndromes. Relatedly, no study to our knowledge has examined how the strength of these associations varies in individuals with syndromic IDDs relative to the general population. It is not known, for example, if economic factors contribute more or less strongly to individual differences in IQ in individuals with syndromic IDDs than they do in the TD population.

#### Recommendation 3: Adopt a common framework organizing relevant social-environmental factors

The field would benefit from a unifying framework identifying the full breadth of relevant SDOH and systemic factors and their specific applications and considerations for individuals living with IDD and their families. This framework would help identify other relevant areas beyond those identified in Figure 3 in which studies of general and clinical populations suggest are linked to cognitive outcomes, including IQ. Examples include exposure to pollutants/toxicants [150], housing stability [151], exposure to community violence [152], discrimination [153], and other areas. Although the theoretical model proposed in the present narrative review highlights a few key SDOH, there is insufficient research on other SDOH factors in individuals with syndromic IDDs to leverage and expand the model beyond its current form. Research examining the impact of SDOH, including expanding our definition and assessment of SDOH factors in research, is needed to more fully understand the etiology of interindividual differences in syndromic IDDs.

The identification of pathways by which these factors impact IQ is crucial to determine which advocacy efforts and interventions may be the most fruitful. For example, the American Association on Intellectual and Developmental Disabilities (AAIDD) regularly publishes a public policy agenda and has published several policy statements informed by this research. One position statement relevant to the present review advocates for significantly increased funding for universal, community-based, early intervention services for young children with or at increased risk for developmental delays [154], which may contribute to gains in IQ and improved quality of life. Our hope is that research clarifying these pathways may help develop a line of evidence-based SDOH-targeted interventions that can be adapted to clinical care.

## Conclusions

Social-environmental factors may explain many interindividual differences in IQ among individuals with syndromic IDDs. Although we discuss evidence of the pathways through which economic instability may influence IQ in this population, there is a dearth of research on the contributions of other social-environmental factors to IQ in individuals with syndromic IDDs. We urge researchers and those who review and publish research (e.g., reviewers and journal editors) to consider the value and importance of incorporating social-demographic information into future work. The addition of this information, especially factors embedded in well-established SDOH frameworks, will support the development of individual-, community-, and systems-level interventions that promote cognitive development and adaptive functioning in individuals with syndromic IDDs.
